# Surgeons’ behaviors and beliefs regarding placebo effects in surgery

**DOI:** 10.1080/17453674.2021.1941627

**Published:** 2021-06-24

**Authors:** Annelie Rosén, Lisbeth Sachs, Amanda Ekdahl, Andreas Westberg, Paul Gerdhem, Ted J Kaptchuk, Karin Jensen

**Affiliations:** a Department of Clinical Neuroscience, Karolinska Institutet, Stockholm, Sweden; b Department of Reconstructive Orthopedics, Karolinska University Hospital, Stockholm, Sweden; c Department of Clinical Science Intervention and Technology, Karolinska Institutet, Stockholm, Sweden; d Department of Orthopedic Surgery, Capio Sankt Göran Hospital, Stockholm, Sweden; e Program in Placebo Studies, Beth Israel and Deaconess Hospital, Harvard Medical School, Boston, MA, USA

## Abstract

Background and purpose — Emerging evidence from sham-controlled trials suggest that surgical treatment entails substantial non-specific treatment effects in addition to specific surgical effects. Yet, information on surgeons’ actual behaviors and beliefs regarding non-specific treatment and placebo effects is scarce. We determined surgeons’ clinical behaviors and attitudes regarding placebo effects.

Methods — A national online survey was developed in collaboration with surgeons and administered via an electronic link.

Results — All surgical clinics in Sweden were approached and 22% of surgeons participated (n = 105). Surgeons believed it was important for them to interact and build rapport with patients before surgery rather than perform surgery on colleagues’ patients (90%). They endorsed the importance of non-specific treatment effects in surgery generally (90%) and reported that they actively harness non-specific treatment effects (97%), including conveying confidence and calm (87%), building a positive interaction (75%), and making eye contact (72%). In communication regarding the likely outcomes of surgery, surgeons emphasized accurate scientific information of benefits/risks (90%) and complete honesty (63%). A majority felt that the improvement after some currently performed surgical procedures might be entirely explained by placebo effects (78%). Surgeons saw benefits with sham-controlled surgery trials, nevertheless, they were reluctant to refer patients to sham controlled trials (46%).

Interpretation — Surgeons believe that their words and behaviors are important components of their professional competence. Surgeons saw the patient–physician relationship, transparency, and honesty as critical. Understanding the non-specific components of surgery has the potential to improve the way surgical treatment is delivered and lead to better patient outcomes.

Accumulating evidence suggests that the elaborate context surrounding surgical treatment may contribute to considerable placebo effects (Kallmes et al. 2009, Beard et al. 2018). Hence, factors other than the surgical intervention itself could contribute to positive health outcomes, and a recent meta-analysis (including 53 trials and 4,000 patients) reported that in 51% of all placebo-controlled surgical trials there was no statistically better result in the surgical arm compared with the placebo arm (Wartolowska et al. 2014b).

Placebo surgery, or sham surgery, is an invasive procedure that has the appearance of a therapeutic intervention, but during which the essential therapeutic maneuver is omitted (Wartolowska et al. 2014). Sham controls can be compared with active treatment in order to elucidate the specific effect of a surgical intervention. Any factors outside the active intervention that affect treatment outcomes can be referred to as non-specific treatment factors. These include, for example, explanations of a treatment (Kam-Hansen et al. 2014), prior experience (Kessner et al. 2013) and the doctor–patient relationship (Kaptchuk et al. 2008).

There is significant evidence that non-specific treatment factors impact treatment outcomes, as demonstrated in many different health problems, using a range of different treatment modalities, including placebo pills, creams, and injections (Finniss et al. 2010, Wager and Atlas 2015). As yet, relatively little is known about non-specific treatment effects and placebos in surgery, and in particular about surgeons’ own behaviors and beliefs. Understanding the non-specific components of surgery has the potential to improve the way surgical treatment is delivered, and lead to better outcomes. The aim of the present study was to describe surgeons’ real-world behaviors and attitudes towards placebo effects. There are no previous studies on placebo attitudes among healthcare professionals in Sweden and in contrast to previous studies (Wartolowska et al. 2014a, Baldwin et al. 2016) the present study reports specific behaviors that surgeons engage in when they harness placebo effects.

## Methods

In a national online survey, Swedish surgeons were asked questions about their clinical behaviors and attitudes regarding non-specific treatment effects and placebo effects.

### Survey preparation

A focus-group meeting was organized to prepare for the survey, including 9 surgeons at the Karolinska University Hospital (8 male, 1 female). A first version of the survey was sent to medical students (n = 11) in order to ensure face validity. To compare our results with other surveys on placebo effects in surgery we adapted some questions to the present study (Tilburt et al. 2008, Raz et al. 2011, Wartolowska et al. 2014a, Baldwin et al. 2016).

### Survey participants

Participants were surgeons, of any surgical specialty, affiliated to a surgical clinic or surgical department in Sweden. Inclusion criteria required that participants were (1) licensed surgeons operating in Sweden, and (2) able to read and understand Swedish. All heads of surgery clinics in Sweden were contacted by email list provided by the Swedish Surgical Society (www.svenskkirurgi.se), a body that organizes surgeons working in Sweden. Heads of surgery were asked if they would be willing to provide the individual email addresses of their surgeons. If yes, each surgeon received an email containing information on the study and a link to the survey.

### Survey procedure

The survey was created in the software Survey& Report version 4.2.33.5 (https://sunet.se). The final survey consisted of 32 questions, including demographics, and took approximately 10 minutes to answer. To minimize conceptual ambiguity, definitions of specific and non-specific treatment effects were included on the first page. A specific treatment effect was defined as “an effect related to the specific medical treatment, i.e., the surgical procedure or a pharmacological substance to relieve symptoms.” A non-specific treatment effect was defined as “an effect related to the context surrounding the delivery of treatment.” In order to facilitate comparisons with previous surveys, we used a wide definition of the placebo effect, i.e., any improvement in response to a placebo, including natural history and regression to the mean. Participants were instructed to answer the questions based on their everyday work in the clinic, as to reflect real-world medical behavior. The survey started with general questions regarding the impact of non-specific treatment effects before introducing more specific (and potentially more controversial) questions regarding sham surgery. A summary of the survey content can be found in Table 1 and a full translation is provided as Supplementary data.

**Table 1.** Survey content: short description of the questions in the national survey to surgeons in Sweden, divided by topic ^a^

The impact of the doctor–patient relationship (patient scenario)
Do you believe that the described scenario may impact the treatment outcome? (Y/N) **^b^**
Non-specific treatment effects in surgery
Do you believe that non-specific treatment effects play a role in surgical treatment? (Y/N)
Which of the following factors do you believe affect treatment outcomes (multiple choice)? ^b^
Do you deliberately harness non-specific treatment effects in treatment of patients? (Y/N)
If yes, which non-specific treatment factors have you used? (multiple choice) ^b^
Framing and communication of treatment outcomes
How would you describe the information you give to your patients regarding expected outcomes of the surgical treatment? (multiple choice) ^b^
Performing surgery that includes placebo components
How often do you perform surgery that you believe includes a placebo component? (multiple choice)
Are there, in your view, surgical treatments that have no specific component, where the treatment outcome is entirely due to the placebo effect? (Y/N) ^b^
Placebo-controlled surgical trials
Sham surgery can only be used if there is no other effective treatment to compare a new treatment with (agree/disagree)
Sham surgery can only be used if there is no risk for adverse events in the placebo group (agree/disagree)
Sham surgery can only be used in trials of non-life-threatening conditions (agree/disagree)
Sham surgery is permissible when there is uncertainty about a specific treatment effect (agree/disagree)
What can, in your view, be problematic when using sham-controlled study designs? (multiple choice) ^b^
Would you personally be able to recruit patients to a sham-controlled surgical trial? (Y/N)
Sham surgery in clinical routine
Are there, in your view, clinical situations when sham surgery might be warranted as it has been proven effective in sham-controlled trials? (Y/N)
Placebo definition, clinical value, and possible mechanisms
Do you agree with the following definition of the placebo effect? (Y/N) ^b^
Do you agree with the following definition of placebo effects in surgery? (Y/N) ^b^
What mechanisms do you believe explain the placebo effect? (multiple choice) ^b^
Do you believe that the placebo effect is true, i.e., has a scientific explanation? (Y/N)
Do you believe that the placebo effect may have a therapeutic benefit? (Y/N)

**^a^**For a full version of the survey, see Supplementary data.

**^b^**Space provided for additional comments.

(Y/N) = Yes or No question; forced choice

The data collection was open between April and September 2018. 2 reminder emails were sent to anyone who did not respond to the initial invitation.

### Ethics, funding, and potential conflicts of interests

Ethical approval for the focus group and survey was obtained from the regional ethical review board in Stockholm, Sweden (Dnr 2018/514-31/1). All surgeons, both in the focus group and survey, gave written informed consent. The authors have no conflicts of interest. The present work was supported by the Pro Futura Grant from the Bank of Sweden Tercentenary Foundation.

## Results

### Demographics

The survey was sent to 478 surgeons and 105 (22%) responded, which is similar to the response rate in a previous survey study among surgeons (Baldwin et al. 2016). None of the 478 emails bounced back due to invalid email addresses, and together with the two reminder emails we hope that the invitations were properly received. According to the National Board of Health and Welfare (Socialstyrelsen 2019) the surgeons who responded to the survey are representative of all Swedish surgeons (listed as specialists in surgery, pediatric, hand, or plastic surgery) in terms of age (median 45–49 years, range 30–70 years) and gender (31% women), except for neurosurgery and thoracic surgery where there are fewer women (21% and 25%, respectively). We have no descriptive information for those who did not respond to our survey. For demographics, see Table 2.

**Table 2.** Demographics ^a^

Mean age (SD) [range]	47 (11) [31–69]
Years since MD license was obtained, mean (SD) [range]	18 (10) [2–40]
Mean number of patients seen/week (SD) [range]	24 (14) [0–60]
Sex (men/women) (%)	68/32
Type of surgical unit (%) City hospital/Small community/Rural setting	62/34/4

**^a^** Basic information regarding the surgeons who responded to the survey (n = 105), obtained via self-report. The “number of patients seen per week” is an approximation of each surgeon’s degree of patient contact.

### Characteristics

The different surgical specialties among the study participants were general surgery (75%), orthopedic surgery (19%), and other (12%). A wide range of surgery types were represented e.g., thoracic surgery, breast surgery, endocrine surgery, cancer surgery, and gastrointestinal surgery.

### Placebo definition

9 out of 10 surgeons reported that they believe the placebo effect is genuine (91%) and has a therapeutic benefit (87%). When asked what mechanisms underlie placebo effects, the top 5 answers were: psychological (99%), physiological (45%), natural history (41%), unexplained factors (32%), and regression to the mean (24%). The survey reflects a wider definition of placebo where all improvements seen among patients treated with placebo are referred to as placebo effects, hence the inclusion of options such as natural history and regression to the mean (see Part 3 in the survey).

### Impact of the doctor–patient relationship

9 out of 10 surgeons (90%) believed that operating on another surgeon’s patient may have an impact on the treatment outcome, i.e., pre-surgical assessments made by one surgeon but surgery performed by an equally skilled colleague. The most common reason for the influence on treatment outcomes is the reduced effect of the doctor–patient relationship (65%), as the interaction between a patient and clinician may build trust prior to the planned surgery (see Part 1 of the survey). In the space for additional comments, one surgeon wrote: “My experience is that any change of doctor involves a risk of problems arising. The established alliance between the patient and doctor is always affected by the change of doctor, as described, and unfortunately often negatively.”

### Non-specific treatment effects in surgery

#### Reported behavior

The survey asked if surgeons’ use of techniques may enhance non-specific treatment effects (see Part 2 of the survey). Almost 9 out of 10 surgeons deliberately use techniques aimed at harnessing non-specific treatment effects (87%). The most common strategy was to “communicate calm and confidence” (76%), followed by “to offer a positive social interaction” (75%), “make eye contact” (72%), “listen with interest” (67%), “look well-groomed” (45%), “confident handshake” (44%), “communicate positive expectations” (41%), and “treatment room clean” (31%). Several choices were allowed (Question 5 in the survey). In the space for additional comments one surgeon wrote: “I use my voice, which is slow with a strong local (northern) accent, I also move slowly in order to create the illusion that we have plenty of time.”

#### Reported attitudes

The survey asked questions about attitudes towards the importance of non-specific treatment effects. A vast majority of surgeons believe that non-specific treatment effects play a role in surgical treatments (97%). Only 3% stated that non-specific treatment factors have no effect in surgical treatments. The top 5 factors that surgeons believe may affect the treatment outcome, in addition to the specific effects of surgical interventions, were (several options possible): patient believes in treatment (91%), the doctor–patient relationship (85%), doctor conveys calm and assertiveness (85%), doctor believes in treatment (82%), the interaction and care from healthcare providers other than the surgeon (79%) (see Part 2 of the survey). In the space for additional comments several surgeons expressed a need to balance high expectations. One stated that: “Above all, I convey realistic and honest expectations. I never oversell.”

### Framing and communication of treatment outcomes

#### Reported behavior

When asked how surgeons would characterize the information they give to patients regarding the possible outcomes of surgery, 9 out of 10 prefer to give what they believe is an accurate description of risks and benefits of the treatment (91%). Information described as “completely honest” was used by 63%, “calming/reducing anxiety” (63%), “hopeful” (33%), and “involves positive expectations” (29%). One quote from the space for additional comments read: “I give the patient realistic expectations, both when it comes to time frames and the result of the operation. If one is too positive it can have the opposite effect”.

### Performing surgery with a placebo component

#### Reported behavior

When asked how often surgeons perform surgery themselves that they believe has a placebo component, there were 5 options ranging from regularly (more than once per week) to never. Half of them agree it is part of their normal practice. 15% say they do it “regularly” (more than once per week), 17% “often” (more than once per month), and 21% “sometimes” (more than once per year). The other half of respondents say they do it “rarely” (less than once per year) (26%) or “never” (23%) (see Part 2 of the survey).

#### Reported attitudes

When asked if surgeons believe that there are surgical treatments where the entire treatment effect is due to placebo, 78% of surgeons said yes. In the space for additional comments surgeons gave examples, e.g., varicose vain surgery, orthopedic surgery (not specified), gallbladder surgery for pain, or hernia surgery. One responded that “it may happen in other countries” (see Part 4 of the survey).

### Placebo-controlled surgical trials

#### Reported behavior

When asked if they would personally be willing to recruit patients to sham-controlled surgical trials, less than half of surgeons said yes (47%) (see Part 4 of the survey).

#### Reported attitudes

When asked about their beliefs towards placebo-controlled clinical trials, 71% responded that placebo-controlled surgical trials can be used when there is uncertainty about the mechanism of an established surgical procedure. 74% responded that placebo should only be used in conditions that are not life-threatening. 71% answered that placebo-controlled surgery should only be used if there are no risks involved, such as general anesthesia. 51% answered that it should only be used if there is no other effective treatment with which to compare the intervention. 33% answered that placebo surgery should only be used in designs where all patients can cross over and get real surgery (see Part 4 of the survey).

When asked for their attitudes towards complications around sham surgery, ethical considerations were most commonly mentioned (88%), followed by the potential side effects, e.g., from general anesthesia (81%). Half of the participants responded that patient’s trust in doctors may be affected negatively (49%). The use of concealment in placebo-controlled trials was mentioned as an obstacle by 40% of surgeons, and the lack of effectiveness of placebo surgery was the response by 35%. When asked about usage of sham surgery in clinical routine, 36% of surgeons find the use of sham surgery permissible as it has been proven effective in sham-controlled trials (see Part 4 of the survey).

## Discussion

We examined whether, and to what extent, surgeons acknowledge and implement non-specific treatment effects in their clinical routine. Additionally, this study examined surgeons’ attitudes towards sham-controlled surgical trials. In contrast to 2 previous survey studies among surgeons (Wartolowska et al. 2014a, Baldwin et al. 2016), our survey assessed specific behaviors that surgeons engage in when harnessing placebo components of surgery, aiming towards a more concrete understanding of surgeons’ practices. Moreover, we asked specific questions about the way surgeons shape their patient information regarding likely outcomes of the surgery. This was a way to address the role of expectations and how they may shape surgery outcomes. To our knowledge, this has not been reported anywhere before. Finally, the present study included concrete questions about surgeons’ willingness to refer patients to sham-controlled surgical trials and found an interesting conflict between what surgeons say and what surgeons are willing to do.

Surgeons in this survey reflect self-awareness and endorse the importance of non-specific treatment effects in surgery, with emphasis on the patient–clinician relationship. Half of the surgeons believe they perform surgical procedures with a placebo component, and deliberately use techniques aimed at harnessing non-specific treatment effects. Thus, surgeons explicitly acknowledge the placebo effect in their own clinical practice. One question is whether surgeons’ attitudes and behaviors can be compared to medical doctors in other medical disciplines. In a study from the UK, 77% of primary care doctors reported using “impure placebos” regularly (at least once per week) (Howick et al. 2013), which means they are using active drugs as placebos (such as treating a viral infection with antibiotics). Similar results were found in the USA, where around half of internists and rheumatologists reported usage of impure placebos (Tilburt et al. 2008).

Our data indicates that surgeons are self-aware and deliberately seek to maximize non-specific treatment effects. In particular, they strive to build a good rapport with their patients, for example by making eye contact, conveying calm, and using attentive listening.

In spite of the strong belief that non-specific components play a central role in surgical treatment, surgeons are generally not fostering positive treatment expectations. Instead, they prefer to use accurate information about potential benefits of surgery when informing their patients. Our data reflects a notion among surgeons where trust is the most important non-specific treatment component, as they prefer to understate the potential of a treatment rather than risk the patient’s trust in the surgeons’ clinical judgment. This is contrary to a commonly repeated opinion suggesting that surgeons overestimate the effects of surgery, and that their judgment of when to operate (or not) is biased (Perezgonzalez 2018); sometimes referred to as the law of the instrument (Kaplan 1964). Furthermore, our data indicates that surgeons prefer to perform surgery on their own patients, rather than colleagues’ patients, so as to maintain the patient–clinician relationship and thereby obtain better surgical outcomes. Surgeons mentioned this as an important part of their professional competence, even if they were not aware of any scientific evidence to support its clinical advantage. Our study suggests that surgeons’ behaviors are directed by strong beliefs regarding non-specific treatment factors—at least their reported behaviors, as we did not measure actual decisions in the surgical clinics. Empirical studies—if ethically feasible—should be performed in order to verify some of these longstanding assumptions.

There have been few previous studies investigating doctors’ attitudes towards placebos in general (Tilburt et al. 2008, Fassler et al. 2010, Howick et al. 2013), and very little about surgery in particular (Campbell et al. 2011, Wartolowska et al. 2014a). As this is the first study that focused on surgeons’ actual behaviors in the clinic, we found that not all reported behaviors were congruent with surgeons’ attitudes. In line with a previous report of surgeons’ attitudes towards sham-controlled clinical trials in the UK (Campbell et al. 2011), our survey indicates that surgeons seek support for surgical procedures from sham-controlled trials. A large majority saw the potential value of comparing real and sham surgery. Nevertheless, less than half of surgeons (47%) were willing to recruit their own patients to sham-controlled trials (the comparable number in the UK trial was 43% [Campbell et al. 2011]). This indicates a conflict between what surgeons think and what they would do. It should be emphasized that positive attitudes towards sham-controlled surgical studies is not the same thing as referring one’s patients to such trials. It is still unclear whether surgeons who were familiar with results from sham-controlled trials were more willing to recruit patients than surgeons with less knowledge about placebo-controlled surgery.

The understanding of surgeons’ attitudes and behaviors may be of importance for predicting advances in surgical medicine. Contrary to assessments of pharmacological treatments, the use of placebo controls is not considered the gold standard in surgery. Based on the results here, and 2 other studies (Campbell et al. 2011, Wartolowska et al. 2014a), it is unlikely that sham-controlled treatment trials—for legitimate ethical, scientific, and feasibility reasons—will become customary in the near future, in spite of surgeons’ understanding of the scientific benefits. In both the Swedish and UK samples, surgeons have concerns about potential side effects from sham surgery and are also apprehensive about the risk to patient–surgeon trust. Yet, there is indication that patients in the placebo arm of sham-controlled studies have less serious adverse events compared with patients in the active treatment arm (Wartolowska et al. 2014b). It is possible that surgeons’ willingness to contribute to sham-controlled trials will change in the future when there is better characterization of the potential risks. In general, a broader discussion of factors that may contribute to patient improvement in surgical trials, including non-specific treatment factors, spontaneous remission, and regression to the mean, will improve the understanding of the specific mechanisms of surgery.

Some surgeons (36%) find it ethically permissible to use sham surgery outside the scope of a clinical trial, and we included this question as the topic was raised in a previous study (Wartolowska et al. 2014a). Using sham surgery as a clinical tool may seem puzzling, but can be compared to so-called “open-label” placebos, an increasing treatment approach where patients are aware they are receiving inactive pills. The popularity (and increased acceptance) of open-label placebos may lead to a shift in attitudes towards inactive treatment in general and lead to clinical applications of sham surgery in the future.

While there are previous reports on the use of placebo treatments in clinical practice (Tilburt et al. 2008, Howick et al. 2013) there is a paucity of data on the comportment physicians adopt in order to engage non-specific treatment effects. The behaviors reported by surgeons here may thus help understand patient–clinician relationships and medical practice in general. As reported here, some behaviors are paradoxical by nature, for example the reluctance among surgeons to induce positive expectations about treatment outcomes. Surgeons explicitly avoid giving positive suggestions regarding treatment outcomes, as maintained trust between patient and clinician is more important for patient outcomes than the potential effect of inducing positive expectations. This indicates a delicate interaction between treatment expectancy and patient–clinician trust that needs to be studied in more detail.

Our findings build on self-reports, and even if surgeons were asked to report real-world behaviors the answers might not be validated objectively. In contrast to questions regarding placebo effects, questions concerning non-specific treatment effects did not explicitly mention natural history or regression-to-the-mean. Thus, it is unclear whether surgeons include these in the concept of non-specific treatment effects. Also, the response rate was only 22%, which may seem low. Yet, the respondents in this study were drawn from the general surgeon population in Sweden, which is a major strength. In contrast, all respondents in the previous survey on placebo attitudes among surgeons (100 respondents) (Wartolowska et al. 2014a) attended a meeting where surgeons were aware of, or involved in, a national sham-controlled trial of shoulder surgery, which restricted the sample. The present sample is representative of surgeons in Sweden belonging to the Swedish Surgical Society and may be generalized within the reasonable limits inherent to survey methodologies in general.

## Supplementary data

Full version of the survey is available as supplementary data in the online version of this article, http://dx.doi.org/10.1080/17453674.2021.1941627

## References

[CIT0001] Baldwin M J, Wartolowska K, Carr A J. A survey on beliefs and attitudes of trainee surgeons towards placebo. BMC Surg 2016; 16(1): 27. 27118280 10.1186/s12893-016-0142-5 PMC4847348

[CIT0002] Beard D J, Rees J L, Cook J A, Rombach I, Cooper C, Merritt N, Shirkey B A, Donovan J L, Gwilym S, Savulescu J, Moser J, Gray A, Jepson M, Tracey I, Judge A, Wartolowska K, Carr A J, Group C S. Arthroscopic subacromial decompression for subacromial shoulder pain (CSAW): a multicentre, pragmatic, parallel group, placebo-controlled, three-group, randomised surgical trial. Lancet 2018; 391(10118): 329–38. 29169668 10.1016/S0140-6736(17)32457-1 PMC5803129

[CIT0003] Campbell M K, Entwistle V A, Cuthbertson B H, Skea Z C, Sutherland A G, McDonald A M, Norrie J D, Carlson R V, Bridgman S, group Ks. Developing a placebo-controlled trial in surgery: issues of design, acceptability and feasibility. Trials 2011; 12:50. 21338481 10.1186/1745-6215-12-50 PMC3052178

[CIT0004] Fassler M, Meissner K, Schneider A, Linde K. Frequency and circumstances of placebo use in clinical practice: a systematic review of empirical studies. BMC Med 2010; 8: 15. 10.1186/1741-7015-8-15 PMC2837612 20178561

[CIT0005] Finniss D, Kaptchuk T, Miller F, Benedetti F. Placebo effects: biological, clinical and ethical advances. Lancet 2010; 375: 686–95. 20171404 10.1016/S0140-6736(09)61706-2 PMC2832199

[CIT0006] Howick J, Bishop F L, Heneghan C, Wolstenholme J, Stevens S, Hobbs F D, Lewith G. Placebo use in the United Kingdom: results from a national survey of primary care practitioners. PLoS One 2013; 8(3): e58247. 23526969 10.1371/journal.pone.0058247 PMC3604013

[CIT0007] Kallmes D F, Comstock B A, Heagerty P J, Turner J A, Wilson D J, Diamond T H, Edwards R, Gray L A, Stout L, Owen S, Hollingworth W, Ghdoke B, Annesley-Williams D J, Ralston S H, Jarvik J G. A randomized trial of vertebroplasty for osteoporotic spinal fractures. N Engl J Med 2009; 361(6): 569–79. 19657122 10.1056/NEJMoa0900563 PMC2930487

[CIT0008] Kam-Hansen S, Jakubowski M, Kelley J M, Kirsch I, Hoaglin D C, Kaptchuk T J, Burstein R. Altered placebo and drug labeling changes the outcome of episodic migraine attacks. Sci Transl Med 2014; 6(218): 218ra5. 10.1126/scitranslmed.3006175 PMC4005597 24401940

[CIT0009] Kaplan A. The conduct of inquiry: methodology for behavioral science. San Francisco: Chandler Publishing; 1964.

[CIT0010] Kaptchuk T J, Kelley J M, Conboy L A, Davis R B, Kerr C E, Jacobson E E, Kirsch I, Schyner R N, Nam B H, Nguyen L T, Park M, Rivers A L, McManus C, Kokkotou E, Drossman D A, Goldman P, Lembo A J. Components of placebo effect: randomised controlled trial in patients with irritable bowel syndrome. BMJ 2008; 336(7651): 999–1003. 18390493 10.1136/bmj.39524.439618.25 PMC2364862

[CIT0011] Kessner S, Wiech K, Forkmann K, Ploner M, Bingel U. The effect of treatment history on therapeutic outcome: an experimental approach. JAMA Intern Med 2013; 173(15): 1468–9. 2013.6705. 23780284 10.1001/jamainternmed.2013.6705

[CIT0012] Perezgonzalez J. Book review: Surgery, the ultimate placebo. Frontiers in Surgery 2018; 5(38): 1–7.29435451

[CIT0013] Raz A, Campbell N, Guindi D, Holcroft C, Dery C, Cukier O. Placebos in clinical practice: comparing attitudes, beliefs, and patterns of use between academic psychiatrists and nonpsychiatrists. Can J Psychiatry/Revue canadienne de psychiatrie 2011; 56(4): 198–208. 21507276 10.1177/070674371105600403

[CIT0014] Socialstyrelsen. http://www.socialstyrelsen.se/statistik/statistikdatabas; 2019.

[CIT0015] Tilburt J C, Emanuel E J, Kaptchuk T J, Curlin F A, Miller F G. Prescribing “placebo treatments”: results of national survey of US internists and rheumatologists. BMJ 2008; 337(oct23_2): a1938-. 18948346 10.1136/bmj.a1938 PMC2572204

[CIT0016] Wager T D, Atlas L Y. The neuroscience of placebo effects: connecting context, learning and health. Nat Rev Neurosci 2015; 16(7): 403–18. 26087681 10.1038/nrn3976 PMC6013051

[CIT0017] Wartolowska K, Beard D J, Carr A J. Attitudes and beliefs about placebo surgery among orthopedic shoulder surgeons in the United Kingdom. PLoS One 2014a; 9(3): e91699. 24632880 10.1371/journal.pone.0091699 PMC3954758

[CIT0018] Wartolowska K, Judge A, Hopewell S, Collins G S, Dean B J, Rombach I, Brindley D, Savulescu J, Beard D J, Carr A J. Use of placebo controls in the evaluation of surgery: systematic review. BMJ 2014b; 348: g3253. 24850821 10.1136/bmj.g3253 PMC4029190

